# Behavioral symptoms among children and adolescents with attention deficit hyperactivity disorder during COVID-19 outbreak: a retrospective prospective cohort study

**DOI:** 10.1186/s43045-022-00198-w

**Published:** 2022-05-05

**Authors:** Nelly R. Abdel Fattah, Amira Mohamed Yousef, Amany Elshabrawy Mohamed, Shimaa Ibrahim Amin

**Affiliations:** 1 Department of Psychiatry, Faculty of Medicine, Zagazig University, Zagazig, Egypt; 2 Faculty of Medicine, Zagazig University, Zagazig, Egypt

**Keywords:** Covid-19, ADHD, Psychological, Behavioral symptoms

## Abstract

**Background:**

Attention deficit hyperactivity disorder (ADHD) is one of the most prevalent neurodevelopmental conditions in children, and with the coronavirus pandemic, ADHD children now pose obvious challenges. This retrospective prospective cohort study was conducted on 150 ADHD children and adolescents that had previously attended the child unit of the Psychiatry Department, Zagazig University Hospitals, Sharkia, Egypt, and diagnosed as ADHD patients using the research diagnostic criteria of DSM-5 which administrated by experienced psychiatrists and evaluated by The Arabic version of Conner’s Parent Rating Scale Revised-short version (CPRS-48) before the onset of COVID-19 pandemic. We collected the other data of the study by applying an Arabic language questionnaire which included the data related to the COVID -19 pandemic and the Arabic version of CPRS-48 by which we reevaluated the behavioral symptoms of the subjects who participated in the study during COVID-19 pandemic. This research aimed to evaluate the behavioral symptoms among ADHD children and adolescents and detect the change in these symptoms during the COVID-19 pandemic by comparing them before and during the pandemic.

**Results:**

One hundred fifty ADHD children were included in the study with a mean age of 10 years old. The male gender was predominant and represented 76.7% of the sample. Ninety percent were living in urban areas with more low social class (50%), 84.7% of parents were married, 60% of the family member of our subjects had COVID-19 while 12% lost one or more of their relative by the pandemic (64.7%). The fathers continued working as normal, while 40.7% of the mothers stopped working because of COVID-19. 62.7% of the parents were verbally and physically abusive to their children. Unfortunately, 100% of the subjects stopped attending their behavioral therapy center, 96.0% stopped their Follow up with a psychiatrist, and 55% stopped complying with their medications. As we presumed, we found a statistically significant change in the behavioral and psychological symptoms measured by Conner’s Parent Rating Scale Revised-short version (CPRS-48) during the COVID-19 pandemic compared to the period before. The worsening of the symptoms was associated with age, sex, residency, social class, father and mother present working and couple status, having positive cases or losses of COVID-19 among family members, and patient maltreatment.

**Conclusions:**

To conclude, this study suggests that the lockdown caused by the COVID-19 pandemic has worsened symptoms in a significant number of children and adolescents with ADHD, which needs clinical attention. Moreover, the patients’ psychiatric follow-up and compliance with their medications were markedly affected. Additionally, the lockdown has also led to an increase in the abusive behavior of the parents towards their children.

## Background

By January 2020, an epidemic of the emerging coronavirus disease COVID-19 had been identified as a Public Medical Crisis of Worldwide Significance. WHO has issued an alert indicating the possibility of COVID-19 spreading to other nations. WHO made the latest declaration that COVID-19 is now a pandemic [1].

COVID-19 is caused by a recently discovered coronavirus that would trigger SARS in man (following zoonotic exposure) [2]. At the onset of the pandemic, most scientific studies have been conducted to clarify better the virus’s characteristics and how to handle it [3].

As the coronavirus pandemic quickly spreads around the globe, it is causing a significant level of anxiety and apprehension, which is more evident in specific communities, including older adults, medical professionals, and people with chronic illnesses, in particular. The COVID-19 pandemic will increase mental health problems among those with pre-existing psychiatric conditions [4].

ADHD is a neurodevelopmental condition characterized by inattention, hyperactivity, and impulsivity. It has a notable impact on an individual’s ability to function and complete daily tasks [5]. 5.3% of the world’s population suffers from this disorder which is one of the most frequent psychiatric conditions throughout infancy [6]. Children with ADHD encounter multiple problems throughout that time of life. The COVID-19 pandemic tremendously influenced every area of their lives to intensify ADHD symptoms [7].

They cannot comprehend the changing circumstances around them from the caregivers’ intimate signals. They cannot be contained to a position and do not access objects they may touch, causing an infection to themselves or their family members. Because of being restricted to one location, ADHD patients are more likely to be anxious and susceptible to impulsive behavior, making it impossible for parents to involve them in constructive activities [8].

The pandemic of COVID-19 is massive, both in nature and magnitude. A fear reaction to coronavirus with the enormous media information which could worsen children’s psychological and behavioral symptoms [9]. Research on normal children and adolescents reported that COVID-19 and its constraint measures had tremendous influences on the emotional state and social interactions [10, 11]. Still, there are great difficulties in explaining the lockdown effect on the behavior and mood of children and adolescents with ADHD due to the following causes. First, the deteriorating mood and behavioral of those patients are usually shown with different severity degrees, and that is independent of the COVID-19 distress; consequently, we may define the impact of constraint measures caused by the pandemic on ADHD patients by the variations in the degree of severity compared to their previous condition. Second, knowing that ADHD patients’ overall functioning dramatically depends on their environmental circumstances [12], we might think their emotional and behavioral differences during COVID-19 could be an essential clue to the change that may occur compared to their previous lifestyle. This study aimed to evaluate the behavioral and psychological symptoms of ADHD children and adolescents, compare them before and during COVID-19, and assess the association between their symptomatology with the sociodemographic and other clinical characteristics.

## Methods

### Study design

A retrospective prospective cohort study was conducted among ADHD children and adolescents who had previously attended the child unit of the Psychiatry Department, Zagazig University Hospitals, Sharkia, Egypt, and were diagnosed as ADHD patients using the research diagnostic criteria of DSM-5 administrated by experienced psychiatrists before the onset of COVID-19 epidemics. The study was performed in the period from May 15 to August 31, 2020.

### Study subjects

One hundred fifty ADHD children and adolescents were selected for this study from the child unit files recorded before COVID-19 pandemics at the hospital mentioned above.

The participants were chosen according to specific criteria as they previously fulfilled DSM-5 criteria for ADHD, with ages ranging from 6 to 17 years old. We included both sex and all socioeconomic classes. Still, we excluded any ADHD cases with an IQ < 90 (which was previously documented in our files) and patients with chronic medical illness or sensory deficit.

### Sampling

To evaluate the behavioral and psychological symptoms of ADHD children and adolescents, compare them before and during COVID-19, the sample size was calculated using power analysis. A minimum total sample size of 147 was sufficient to detect an effect size of 0.3 at a power of 0.95%, a confidence level of 95% at a significance level of 0.05. The sample size was calculated using G*Power software version Version 3.1.9.6 [13]. A total sample size of 150 patients was applied. The sample size was also confirmed with The OPEN EPI software package used to calculate our sample [14]. The sample size was raised by 10% to compensate for the expected dropout.

### Study tools and steps

First, patients’ data were collected retrospectively from patients’ files at the child unit of the psychiatry department at Zagazig University Hospitals which were previously diagnosed as ADHD patients using the research diagnostic criteria of DSM-5, which was administrated by experienced psychiatrists and evaluated by The Arabic version of Conner’s Parent Rating Scale Revised-short version (CPRS-48) before the onset of COVID-19 pandemic. Also, these data included their social status, which was previously evaluated by the scoring system of Fahmy and El-Sherbini for measurement of socioeconomic status [15].

Second, we contacted the parents or caregivers of eligible ADHD patients to inform them about the goal of the study and the techniques. Those who accepted to participate in our study were then sent a message outlining the details of the study. This was a closed trial, with participants drawn from a specific sample of ADHD patients identified personally by the research team. The researcher distributed an electronic Google form to the participants that contained the entire questionnaire, which was then gathered on an excel form for analysis. Just the research team had access to the participants’ identities that were concealed throughout data processing. The electronic google form consisted of three parts:

1. The first part: it included the aim of our study, its procedures and a binary “yes” or “no” inquiry showing the participants’ acceptance or refusal to be involved in our research
2. The second part: included an Arabic language questionnaire. It consists of questions related to sociodemographic data, marital status of the parents, and COVID-19 infection or related fatalities among family members. Parent’s present working status, the availability of face–to–face behavioral therapy center, the consultation with the child’s psychiatrist during the pandemic, and the pharmacological treatment for behavior problems if present and the compliance with it. Parent’s response towards the patient’s behavior problems.
3. The third part: included the Arabic version of Conner’s Parent Rating Scale Revised-short version (CPRS-48). The reevaluation of the behavioral symptoms of the subjects who participated in the study was done. This brief assessment form is composed of subscales that measure (a) conduct difficulties, (b) learning difficulties, (c) psychosomatic difficulties, (d) impulsive–hyperactivity, and (e) anxiety, as well as (f) the item hyperactivity index. The parent(s) graded their children’s behavior throughout the preceding month on 4-point ratings ranging from zero (no problems) to three (severe problems). Typically, this scale takes about half an hour to complete. Each question’s score is transformed into a *t* score with a mean of 50 and a standard deviation of 10. If the child’s *t* scores are two standard deviations above the average, they have a particular behavior difficulty [16]. The national institute of mental health established the validity, reliability, and stability of this measure. The reliability of test-retest and inter-rater comparisons were (0.64 and 0.68), correspondingly [17].

Three psychiatry department specialists evaluated the questionnaire’s content validity. These specialists evaluated the questionnaire’s coherence, relevancy, and comprehensiveness. A pilot study was done 1 month preceding data gathering to detect any challenges in responding to the questionnaire and to assess its reliability. Because of the modifications in the final version of the questionnaire, the pilot research sample was omitted from the overall sample.

### Statistical analysis

SPSS software version 27 was used for statistical analysis [18]. Tables were used to present the data. The mean and standard deviation were used to show quantitative data. Frequencies and proportions were used to portray qualitative data. To establish variable distribution features and variance homogeneity, the Kolmogorov-Smirnov and Levene tests were utilized. The paired *t* test is used to assess paired data.

To assess qualitative variables, Pearson’s chi-squared test and chi-square for linear trend were utilized as needed. As appropriate, the Student *t* test and the Mann-Whitney *U* test were employed to compare quantitative variables between the two groups. When relevant, one-way ANOVA and the Kruskal-Wallis test compared quantitative variables between more than two groups. A *P* value of 0.05 was considered statistically significant.

## Results

Table 1 revealed that the mean age of the participants was 10 years old. The male gender was predominant and represented 76.7% of the sample. Ninety percent were living in urban areas with more low social class (50%), 84.7% of parents were married, 60% of the family member of our subjects had COVID-19, while 12% lost one of their relatives by the epidemic. Twelve percent of the fathers and 40.7% of the mothers stopped working because of COVID-19. 62.7% of the parents were verbally and physically abusive towards their children’s behavior problems compared to before COVID-19. Unfortunately, 100% of the subjects stopped attending their behavioral therapy center, and 74.1% stopped taking their medications. While 96% of the ADHD patients did not have a direct follow-up with their psychiatrist since COVID-19.

**Table 1:** General characteristics of the study participants

Variables	Study participants (***n*** = 150)	Chi-square	
		Chi-	Sign.
Age of child (years): Mean ± SD	10.3 ± 3.1	8.07	0.707 ns
Sex, *n* (%):			
Male	115 (76.7%)	13.89	< 0.001**
Female	35 (23.3%)		
Residence, *n* (%):			
Urban	135 (90.0%)	63.04	< 0.001**
Rural	15 (10%)		
Social class, *n* (%):			
Low	75 (50.0%)	60.20	< 0.001**
Middle	52 (34.7%)		
High	23 (15.3%)		
Marital state of the parents, *n* (%):			
Married	127 (84.7%)	96.24	< 0.001**
Divorced	14 (9.3%)		
Widow	9 (6.0%)		
The number of other siblings, *n* (%):			
Median (range)	2 (1–4)	20.47	< 0.001**
Confirmed positive cases of COVID-19 among family members, *n* (%):			
Yes	90 (60.0%)	8.49	< 0.001**
No	60 (40.0%)		
Lost family member due to COVID-19, *n* (%):			
Yes	18 (12.0%)	72.98	< 0.001**
No	132 (88.0%)		
Father’s current working state, *n* (%):			
Go to work as regular	97 (64.7%)	79.51	< 0.001**
Online working from home	32 (21.3%)		
Not working because of COVID-19	18 (12.0%)		
Not working since before COVID-19	3 (2.0%)		
Mother’s current working state, *n* (%):			
Not working because of COVID-19	61 (40.7%)	20.69	< 0.001**
Not working since before COVID-19	41 (27.3%)		
Online working from home	30 (20.0%)		
Go to work as regular	18 (12.0%)		
Direct behavioral therapy’s center support since COVID-19, *n* (%):			
Yes	0 (0.0%)	–	> 0.05 ns
No	150 (100%)		
Direct follow-up with a psychiatrist since COVID-19, *n* (%):			
Yes	6 (4.0%)	56.82	< 0.001**
No	144 (96.0%)		
The compliance of the ADHD children on treatment as compared to before COVID-19:			
Compliant	39 (25.9%)	4.26	0.039*
Non-compliant	111 (74.1%)		
The parent’s managements of the child’s behavior problems as compared to before COVID-19, *n* (%):			
Not abusive	26 (17.3%)	35.16	< 0.001**
Verbally abusive	30 (20.0%)		
Both verbally and physically abusive	94 (62.7%)		

*significant, **highly siginificant

Table 2 demonstrated a statistically significant difference in the (CPRS-48) scores before and during COVID-19 pandemic in all the subscales.

**Table 2:** Comparison of Conner’s Parent Rating Scale Revised-short version (CPRS-48) scores before and during COVID-19 pandemic

Items	Before COVID-19	During COVID-19	***P***
Conduct score:			
Mean ± SD	58.2 ± 8.0	59.3 ± 8.7	< 0.001**
Average	96 (64.0%)	84 (56.0%)	
High	54 (36.0%)	66 (44.0%)	
Learning score:			
Mean ± SD	64.9 ± 3.5	65.1 ± 4.4	< 0.001**
Average	100 (66.7%)	29 (36.7%)	
High	50 (33.3%)	121 (63.3%)	
Psychosomatic score:			
Mean ± SD	54.4 ± 4.6	58.1 ± 6.3	< 0.001**
Average	135 (90.0%)	98 (65.3%)	
High	15 (10.0%)	52 (34.3%)	
Impulsive hyperactive score:			
Mean ± SD	61.4 ± 7.9	62.8 ± 8.4	< 0.001**
Average	68 (45.3%)	53 (35.3%)	
High	82 (54.7%)	97 (64.7%)	
Anxiety score:			
Mean ± SD	55.6 ± 5.9	61.1 ± 5.9	< 0.001**
Average	109 (72.7%)	56 (37.3%)	
High	41 (27.3%)	94 (62.7%)	
10-items hyperactivity index:			
Mean ± SD	66.6 ± 1.8	66.8 ± 2.9	0.016*
Average	90 (60%)	46 (30.6%)	
High	60 (40%)	104 (69.4%)	

*significant, **highly siginificant

Table 3 indicated that during the pandemic, scores on the conduct, learning, psychosomatic, impulsive-hyperactive, anxiety, and 10-item hyperactivity index subscales increased by 12%, 13.3%, 24.7%, 14%, 38.7%, and 15.3% of ADHD patients, respectively.

**Table 3:** Change in the Conner’s Parent Rating Scale Revised-short version (CPRS 48) scores during COVID-19 pandemic

Conner’s 3rd Edition (PARENT Short Form)	Change after COVID-19 pandemic			***P***
	Increased	No change	Decreased	
Conduct score	18 (12.0%)	126 (84.0%)	6 (4.0%)	0.02*
Learning score	20 (13.3%)	124 (82.7%)	6 (4.0%)	0.01*
Psychosomatic score	37 (24.7%)	113 (75.3%)	0 (0.0%)	< 0.001**
Impulsive hyperactive score	21 (14.0%)	123 (82.0%)	6 (4.0%)	0.01*
Anxiety score	58 (38.7%)	87 (58.0%)	5 (3.3%)	< 0.001**
10-items hyperactivity index	23 (15.3%)	118 (78.7%)	9 (6.0%)	0.03*

*significant, **highly siginificant

Table 4 revealed a statistically significant association between the increase in the conduct behavior score and some factors like having family members infected with coronavirus or deaths related to it. Not working fathers because of COVID-19, an absence of psychiatric follow-up, and the presence of physical and verbal maltreatment of the child.

**Table 4:** Association between the conduct, learning, and psychosomatic score of the ADHD patients during COVID-19 pandemic and their general characteristics

Variables	Conduct score			Learning score			Psychosomatic score		
	Average (***n*** = 84)	High (***n*** = 66)	***P***	Average (***n*** = 29)	High (***n*** = 121)	*P*	Average (***n*** = 98)	High (***n*** = 52)	*P*
Age of child (years):									
Mean ± SD	9.7 ± 2.6	11.0 ± 3.5	0.02*	10.3 ± 2.2	10.3 ± 3.2	0.9	10.8 ± 3.1	9.2 ± 2.6	0.002*
Sex, *n* (%):									
Male	52 (61.9%)	63 (95.5%)	< 0.001*	23 (79.3%)	92 (76.0%)	0.7	89 (90.8%)	26 (50.0%)	< 0.001**
Female	32 (38.1%)	3 (4.5%)		6 (20.7%)	29 (24.0%)		9 (9.2%)	26 (50.0%)	
Residence, *n* (%):									
Urban	78 (92.9%)	57 (86.4%)	0.2	29 (100%)	106 (87.6%)	0.04*	86 (87.8%)	49 (94.2%)	0.2
Rural	6 (7.1%)	9 (13.6%)		0 (0.0%)	15 (12.4%)		12 (12.2%)	3 (5.8%)	
Social class, *n* (%):									
Low	30 (35.7%)	45 (68.2%)	< 0.001*	14 (48.3%)	61 (50.4%)	0.5	54 (55.1%)	21 (40.4%)	0.6
Middle	32 (38.1%)	20 (30.3%)		8 (27.6%)	44 (36.4%)		21 (21.4%)	31 (59.6%)	
High	22 (26.2%)	1 (1.5%)		7 (24.1%)	16 (13.2%)		23 (23.5%)	0 (0.0%)	
Marital state of the parents, *n* (%):									
Married	79 (94.0%)	48 (72.7%)	< 0.001*	29 (100%)	98 (81.0%)	0.04*	77 (78.6%)	50 (96.2%)	0.01*
Divorced	5 (6.0%)	9 (13.6%)		0 (0.0%)	14 (11.6%)		12 (12.2%)	2 (3.8%)	
Widow	0 (0.0%)	9 (13.6%)		0 (0.0%)	9 (7.4%)		9 (9.2%)	0 (0.0%)	
The number of other siblings:									
Median (range)	2 (0 – 4)	1 (0–4)	0.1	2 (0 – 4)	2 (0–4)	0.5	2 (0–4)	2 (0–3)	0.2
Confirmed positive cases of COVID-19 among family members, *n* (%):									
Yes	39 (46.4%)	51 (77.3%)	< 0.001*	12 (41.4%)	78 (64.5%)	0.02*	63 (64.3%)	27 (51.9%)	0.1
No	45 (53.6%)	15 (22.7%)		17 (58.6%)	43 (35.5%)		35 (35.7%)	25 (48.1%)	
Lost family member due to COVID-19, *n* (%):									
Yes	6 (7.1%)	12 (18.2%)	<0.001*	0 (0.0%)	18 (14.9%)	0.03*	12 (12.3%)	6 (11.5%)	0.6
No	78 (92.9%)	54 (81.8%)		29 (100%)	103 (85.1%)		86 (87.8%)	46 (88.5%)	
Father’s current working state, *n* (%):									
Go to work as regular	49 (58.3%)	48 (72.7%)	0.04*	15 (51.7%)	82 (67.8%)	0.3	63 (64.3%)	34 (65.4%)	0.001**
Online working from home	23 (27.4%)	9 (13.6%)		8 (27.6%)	24 (19.8%)		17 (17.3%)	15 (28.8%)	
Not working because of COVID-19	9 (10.7%)	9 (13.6%)		6 (20.7%)	12 (9.9%)		18 (18.4%)	0 (0.0%)	
Not working since before COVID-19	3 (3.6%)	0 (0.0%)		0 (0.0%)	3 (2.5%)		0 (0.0%)	3 (5.8%)	
Mother’s current working state, *n* (%):									
Not working because of COVID-19	34 (40.5%)	27 (40.9%)	< 0.001*	14 (48.3%)	47 (38.8%)	< 0.001*	35 (35.7%)	26 (50.0%)	0.2
Not working since before COVID-19	20 (23.8%)	21 (31.8%)		0 (0.0%)	41 (33.9%)		27 (27.6%)	14 (26.9%)	
Online working from home	27 (32.1%)	3 (4.5%)		15 (51.7%)	15 (12.4%)		21 (21.4%)	9 (17.3%)	
Go to work as regular	3 (3.6%)	15 (22.7%)		0 (0.0%)	18 (14.9%)		15 (15.3%)	3 (5.8%)	
Direct follow-up with a psychiatrist since COVID-19, *n* (%):									
Yes	6 (7.1%)	0 (0.0%)	0.03*	3 (10.3%)	3 (2.5%)	0.05*	6 (6.1%)	0 (0.0%)	0.07
No	78 (92.9%)	66 (100%)		26 (89.7%)	118 (97.5%)		92 (93.9%)	52 (100%)	
The compliance of the ADHD children on treatment as compared to before COVID-19:									
Compliant	29 (44%)	9 (10.7%)	0.004*	15 (51.7%)	26 (21.4%)	0.017*	59 (60%)	18(34.6%)	0.06
Non-compliant	37(56%)	75 (89.3%)		14 (48.3%)	95 (78.2%)		39 (40%)	34 (65.4%)	
The parent’s attitude towards the child’s behavior problems as compared to before COVID-19, *n* (%):									
Not abusive	17 (20.2%)	9 (13.6%)	< 0.001*	3 (10.3%)	23 (19.0%)	0.09	6 (6.1%)	20 (38.4%)	< 0.001**
Verbally abusive	28 (33.3%)	2 (3.0%)		3 (10.3%)	27 (22.3%)		18 (18.4%)	12 (23.1%)	
Both verbally and physically abusive	39 (46.4%)	55 (83.3%)		23 (79.4%)	71 (55.7%)		74 (75.5%)	20 (38.5%)	

*significant, **highly siginificant

It also showed the association between increased learning problem scores and COVID-19 cases or deaths among the family members and the absence of psychiatric follow-up. Moreover, it demonstrated that the increased psychosomatic scores were statistically associated with the younger age of the child, male sex, married parents, working status of the father, and not abusive parents.

Table 5 revealed that the increased impulsive-hyperactive scores were associated with male sex and grief due to COVID-19. At the same time, the increased anxiety and 10-items hyperactivity index score were associated with the younger age of the child, male sex, divorced parents, and the working status of the father.

**Table 5:** Association between the impulsive hyperactive, anxiety, and 10-items hyperactivity index score of ADHD patients during COVID-19 pandemic and their general characteristics

Variables	Impulsive hyperactive score			Anxiety score			10-items hyperactivity index		
	Average (***n*** = 53)	High (***n*** = 97)	***P***	Average (***n*** = 56)	High (***n*** = 94)	*P*	Average (***n*** = 46)	High (***n*** = 104)	*P*
Age of child (years):									
Mean ± SD	10.4 ± 2.6	10.2 ± 3.3	0.6	10.9 ± 3.4	9.9 ± 2.8	0.04*	11.1 ± 2.6	9.9 ± 3.2	0.02*
Sex, *n* (%):									
Male	21 (39.6%)	94 (96.9%)	< 0.001*	56 (100%)	59 (62.8%)	< 0.001*	26 (56.5%)	89 (85.6%)	< 0.001**
Female	32 (60.4%)	3 (3.1%)		0 (0.0%)	35 (37.2%)		20 (43.5%)	15 (14.4%)	
Residence, *n* (%):									
Urban	47 (88.7%)	88 (90.7%)	0.7	50 (89.3%)	85 (90.4%)	0.8	43 (93.5%)	92 (88.2%)	0.3
Rural	6 (11.3%)	9 (9.3%)		6 (10.7%)	9 (9.6%)		3 (6.5%)	12 (11.5%)	
Social class, *n* (%):									
Low	25 (47.2%)	50 (51.5%)	0.6	31 (55.4%)	44 (46.8%)	0.4	25 (54.3%)	50 (48.1%)	0.1
Middle	21 (39.6%)	31 (32.0%)		19 (33.9%)	33 (35.1%)		11 (24.0%)	41 (39.4%)	
High	7 (13.2%)	16 (16.5%)		6 (10.7%)	17 (18.1%)		10 (21.7%)	13 (12.5%)	
Marital state of the parents, *n* (%):									
Married	48 (90.6%)	79 (81.4%)	0.07	44 (78.6%)	83 (88.3%)	< 0.001*	44 (95.7%)	83 (79.8%)	0.04*
Divorced	5 (9.4%)	9 (9.3%)		3 (5.4%)	11 (11.7%)		2 (4.3%)	12 (11.5%)	
Widow	0 (0.0%)	9 (9.3%)		9 (16.1%)	0 (0.0%)		0 (0.0%)	9 (8.7%)	
The number of other siblings									
Median (range)	2 (0–4)	2 (0–4)	0.9	2 (0–4)	1 (0–3)	0.8	2 (0–3)	2 (0–4)	0.4
Confirmed positive cases of COVID-19 among family members, *n* (%):									
Yes	27 (50.9%)	63 (64.9%)	0.09	33 (58.9%)	57 (60.6%)	0.8	24 (52.2%)	66 (63.5%)	0.2
No	26 (49.1%)	34 (35.1%)		23 (41.1%)	37 (39.4%)		22 (47.8%)	38 (36.5%)	
Lost family member due to COVID-19, *n* (%):									
Yes	0 (0.0%)	18 (18.6%)	< 0.001*	9 (16.1%)	9 (9.6%)	0.5	3 (6.5%)	15 (14.4%)	0.04*
No	53 (100%)	79 (81.4%)		47 (83.9%)	85 (90.4%)		43 (93.5%)	89 (85.6%)	
Father’s current working state, *n* (%):									
Go to work as regular	32 (60.4%)	65 (67.0%)	0.3	36 (64.3%)	61 (64.9%)	0.003*	26 (56.5%)	71 (68.3%)	0.2
Online working from home	15 (28.3%)	17 (17.5%)		8 (14.3%)	24 (25.5%)		14 (30.4%)	18 (17.3%)	
Not working because of COVID-19	6 (11.3%)	12 (12.4%)		12 (21.4%)	6 (6.4%)		6 (13.0%)	12 (11.5%)	
Not working since before COVID-19	0 (0.0%)	3 (3.1%)		0 (0.0%)	3 (3.2%)		0 (0.0%)	3 (2.9%)	
Mother’s current working state, n (%):									
Not working because of COVID-19	20 (37.7%)	41 (42.3%)	0.3	23 (41.1%)	38 (40.4%)	0.9	22 (47.8%)	39 (37.5%)	0.2
Not working since before COVID-19	12 (22.6%)	29 (29.9%)		15 (26.8%)	26 (27.7%)		9 (19.6%)	32 (30.8%)	
Online working from home	15 (28.3%)	15 (15.5%)		12 (21.4%)	18 (19.1%)		12 (26.1%)	18 (17.3%)	
Go to work as regular	6 (11.3%)	12 (12.4%)		6 (10.7%)	12 (12.8%)		3 (6.5%)	15 (14.4%)	
Direct follow-up with a psychiatrist since COVID-19, *n* (%):									
Yes	3 (5.7%)	3 (3.1%)	0.3	3 (5.4%)	3 (3.2%)	0.5	3 (6.5%)	3 (2.9%)	0.3
No	50 (94.3%)	94 (96.9%)		53 (94.6%)	91 (96.8%)		43 (93.5%)	101 (97.1%)	
The compliance of the ADHD children on treatment as compared to before COVID-19:									
Compliant	58 (59.7%)	16 (30.1%)	0.032*	47 (84%)	48 (51.1%)	0.006*	28 (60.8%)	32 (30.7%)	0.032*
Non-compliant	39 (40.3%)	37 (69.8%)		9 (16%)	46 (48.9%)		18 (39.2%)	72 (69.3%)	
The parent’s attitude towards the child’s behavior problems as compared to before COVID-19, *n* (%):									
Not abusive	9 (17.0%)	17 (17.6%)	0.1	0 (0.0%)	26 (27.6%)	< 0.001*	3 (6.5%)	23 (22.1%)	0.9
Verbally abusive	18 (34.0%)	12 (12.4%)		6 (10.7%)	24 (25.5%)		18 (39.1%)	12 (11.5%)	
Both verbally and physically abusive	26 (49.0%)	68 (70.0%)		50 (89.3%)	44 (46.9%)		25 (54.4%)	69 (66.4%)	

*significant, **highly siginificant

## Discussion

COVID-19 is produced by a recently discovered coronavirus [19]. It was observed for the first time in December 2019 in Central China, from the first day the outbreak began, most clinical and scientific work has been directed toward increasing our knowledge about the virus’ characteristics and pathogenic arsenal to cure and prevent infection [3]. However, some research indicates that the pandemic exposes a possible shortage in emergency psychiatric care [20]. The COVID-19 epidemic, in particular, would result in increased emotional distress among the general populace and an increased risk of or worsening of pre-existing mental health conditions [3].

Among vulnerable groups, young people with ADHD are of significant concern because of the COVID-19 pandemic’s possible effects and the additional help they might require maintaining their mental well-being throughout the pandemic [9].

To our knowledge, our study is one of a few studies in Egypt to examine the effect of COVID-19 on a sensitive population of ADHD patients, to compare their behavior to the period before COVID-19. This research established a link between the time of transition and constraint associated with COVID-19 and a worsening of ADHD symptoms in these individuals during the pandemic, most significantly, in the psychosomatic and anxiety symptoms. Additionally, several children maintained their issues at the same level, and only a small number of children made any improvement toward resolving their behavioral concerns during the constraint time. ADHD individuals have a poor capacity for adapting to uncertainty, which makes following instructions and interpreting the overall situation difficult. According to Cortese et al. 2020, the pressured atmosphere at home and the hostile surroundings, both of which disturb young children’s typical rhythm, enhance the chance of much more significant hyperactive and impulsive problems. Furthermore, the nature of the disorder itself contributes to the worsening of symptoms [21].

In accordance with our results, Zhang et al. [8] observed that ADHD symptoms deteriorated significantly during the coronavirus outbreak when relative to their pre-pandemic condition. Additionally, Shah R. et al. [22] discovered that ADHD symptomatology had deteriorated in half of their participating subjects during the lockout caused by the pandemic manifesting as an increase in the level of activity, irritability, or conduct symptoms. We must put into consideration that the instability in emotion and decreased adaptive functioning are significantly related to the primary symptomatology of ADHD patients [23–25].

Although Bobo et al., in their study, discovered identified decreases in restlessness in relation to the lowering of anxiety caused by the enforced routine of educational activity with less academic stress and routines that corresponded to the children’s cycles. Also, the improved self-esteem of the children as a result of receiving less unfavorable remarks, while other parents reported a worsening of oppositional behavior, emotional outbursts, and insomnia [26].

Unfortunately, all of our participants stopped attending their behavioral therapy center and 74.1% were not compliant with their medications. While 96% of the ADHD patients did not have a direct follow-up with their psychiatrist since COVID-19. In the study of Shah R. et al., which detected the effect of the pandemic and its measures on the patients and their families, they documented that more than half of parents reported having problems obtaining prescriptions for their children affecting their children’s compliance [22]. This was a significant catastrophic drawback of the pandemic on those patients, which needs extensive attention and care to help this group of patients retain their compliance and improve their symptoms. We must balance the safety of our patients on one side and their mental health on the other. We think that ADHD patients adapt better to brief lockdowns; Waite P et al. found that children placed in lockdown for 3 months exhibited more severe behavioral problems [27].

Moreover, we revealed that 62.7% of the parents were verbally and physically abusive towards their children’s behavior problems compared to the pre-pandemic state. The study by Shah R. et al. [22] reported that many parents reported an increase in their negative emotions and undesirable behaviors towards their children, such as impatience, yelling at the kid, slapping, verbal abuse, and disciplining their children [19]. We think that this may represent the disorder’s nature in that if the parents are obligated to spend more hours with the children and are forced to take care of their children 24/7 during the time of constraint caused by the pandemic, they may become weary and respond negatively to them. It is critical to give psychoeducation to parents, who should alternate managing ADHD patients and have a pause or rest to control their feelings and actions. ADHD patients have significant difficulties with their social, cognitive, and academic performance. These patients have to comply with their medications to manage these difficulties [28], which might affect the individuals’ and families’ well-being [29]. To be a parent of an ADHD patient is a demanding and challenging job. Handling the psychosocial problems of their children make them feel unqualified, which harm their psychological state [30]. Dealing with such a patient during a stressful environment of COVID-19 added tremendous pressure on their families who have to deal with the pandemic drawbacks, among them the financial impact, the losing jobs and the fear of being infected or losing loved ones, all of contribute to the stress of those parents which may significantly affect the way they are managing their children’ behavior.

## Limitation of the study

We could not conduct a formal psychiatric interview due to the lockdown measures with our patients. Also, some of our data were obtained subjectively by the parents rather than applying a specific questionnaire directly to the children. Moreover, we could not investigate additional aspects, like familial relationships, other pressures, and parental personal traits, which may affect the improvement or deterioration of our patient’s symptoms.

## Conclusions

To conclude, according to this study, the lockdown induced by the COVID-19 outbreak had aggravated symptoms in a considerable proportion of children and adolescents with ADHD that requires professional care. Furthermore, the patients’ psychiatric follow-up and medication adherence were significantly impacted. Moreover, the lockdown has resulted in a rise in aggressive behavior by parents against their children.

## Data Availability

All the data are included in the study.

## Abbreviations

ADHD: Attention deficit hyperactivity disorder
CPRS-48: Conner’s Parent Rating Scale Revised-short version
COVID-19: Coronavirus disease of 2019
WHO: World Health Organization
SARS: Severe acute respiratory syndrome
